# The MEK-ERK pathway negatively regulates *bim *expression through the 3' UTR in sympathetic neurons

**DOI:** 10.1186/1471-2202-12-69

**Published:** 2011-07-15

**Authors:** Rosie Hughes, Jonathan Gilley, Mark Kristiansen, Jonathan Ham

**Affiliations:** 1 Molecular Haematology and Cancer Biology Unit, Institute of Child Health, University College London, 30 Guilford Street, London, WC1N 1EH, UK; 2 Laboratory of Signalling and Cell Fate, The Babraham Institute, Cambridge, CB22 3AT, UK

## Abstract

**Background:**

Apoptosis plays a critical role during neuronal development and disease. Developing sympathetic neurons depend on nerve growth factor (NGF) for survival during the late embryonic and early postnatal period and die by apoptosis in its absence. The proapoptotic BH3-only protein Bim increases in level after NGF withdrawal and is required for NGF withdrawal-induced death. The regulation of Bim expression in neurons is complex and this study describes a new mechanism by which an NGF-activated signalling pathway regulates *bim *gene expression in sympathetic neurons.

**Results:**

We report that U0126, an inhibitor of the prosurvival MEK-ERK pathway, increases *bim *mRNA levels in sympathetic neurons in the presence of NGF. We find that this effect is independent of PI3-K-Akt and JNK-c-Jun signalling and is not mediated by the promoter, first exon or first intron of the *bim *gene. By performing 3' RACE and microinjection experiments with a new *bim*-LUC+3'UTR reporter construct, we show that U0126 increases *bim *expression via the *bim *3' UTR. We demonstrate that this effect does not involve a change in *bim *mRNA stability and by using PD184352, a specific MEK1/2-ERK1/2 inhibitor, we show that this mechanism involves the MEK1/2-ERK1/2 pathway. Finally, we demonstrate that inhibition of MEK/ERK signalling independently reduces cell survival in NGF-treated sympathetic neurons.

**Conclusions:**

These results suggest that in sympathetic neurons, MEK-ERK signalling negatively regulates *bim *expression via the 3' UTR and that this regulation is likely to be at the level of transcription. This data provides further insight into the different mechanisms by which survival signalling pathways regulate *bim *expression in neurons.

## Background

In mammalian cells, two major apoptotic pathways have been described: the extrinsic (death receptor) pathway and the intrinsic (mitochondrial) pathway. The permeabilisation of the mitochondrial outer membrane is a key step in the intrinsic pathway, which proceeds following complex interactions between proapoptotic and antiapoptotic members of the Bcl-2 superfamily of cell death regulators. The BH3-only Bcl-2 family protein Bim is an important initiator and regulator of the intrinsic pathway since Bim can interact with both the antiapoptotic Bcl-2 proteins and the multidomain proapoptotic effector proteins Bax and Bak [1,2].

Bim is a critical mediator of apoptosis in many cell types including NGF-dependent sympathetic neurons. In these cells *bim *RNA and Bim protein levels increase rapidly following NGF deprivation and peak at around 16 hours later [3,4]. Overexpression of Bim_EL _in sympathetic neurons is sufficient to induce the release of cytochrome *c *and apoptosis in the presence of NGF and sympathetic and sensory neurons isolated from *bim*^-/- ^knockout mice are significantly protected from trophic factor withdrawal-induced death [3-5].

The binding of NGF to the TrkA tyrosine kinase receptor on the surface of sympathetic neurons activates the PI3-K-Akt and Raf-MEK-ERK signalling pathways which can both inhibit apoptosis and promote cell survival [6,7]. It is now evident that a number of regulatory mechanisms exist to prevent the inappropriate expression of *bim *in neurons. Three transcriptional pathways have been described, each of which targets elements within the *bim *promoter. Firstly, *bim *transcription is repressed by PI3-K-Akt signalling: active Akt phosphorylates FOXO3a, which is sequestered in the cytoplasm by 14-3-3 protein and following NGF withdrawal FOXO3a translocates into the nucleus of sympathetic neurons and activates *bim *expression via two conserved FOXO binding sites [8,9]. Secondly, *bim *is activated by a Cdk4-E2F-Myb pathway following NGF withdrawal in neuronally differentiated PC12 cells and this requires Myb binding sites in the *bim *promoter [10]. Thirdly, MLK-JNK-c-Jun signalling appears to be critical for *bim *upregulation in sympathetic neurons: the overexpression of a dominant negative c-Jun protein reduces the increase in *bim *mRNA and protein level that occurs after NGF withdrawal [3] and the *jun^AA ^*knock-in mutation, which eliminates the two major JNK phosphorylation sites in c-Jun, reduces the increase in Bim protein level after NGF withdrawal [11].

Post-translational mechanisms that regulate the activity of the Bim protein in neuronal cells have also been described. Bim_EL _is a target of the RAF-MEK-ERK signalling pathway, which delivers important survival signals in many different cell types. In neuronally differentiated PC12 cells maintained in the presence of NGF, Bim_EL _is phosphorylated by ERK [12], and studies with serum-treated fibroblasts have demonstrated that Bim_EL _contains an ERK1/2 docking site and ERK phosphorylation sites via which the MEK1/2-ERK1/2 pathway promotes the phosphorylation of Bim_EL _leading to its ubiquitylation and degradation via the proteasome [13-17]. Furthermore, the ERK1/2-dependent phosphorylation of Bim_EL _leads to the dissociation of Bim_EL _from complexes with the antiapoptotic Mcl-1 and Bcl-xL proteins and also the proapoptotic Bax protein [17,18].

However, it is not known whether the MEK-ERK pathway controls *bim *expression in neurons at a step prior to the phosphorylation of Bim_EL_. Here, we have investigated this using NGF-dependent developing sympathetic neurons. We show that in the presence of NGF, MEK-ERK signalling reduces *bim *mRNA levels and that this is a transcriptional mechanism mediated through the *bim *3' UTR.

## Results

### The MEK-ERK pathway negatively regulates *bim *mRNA expression independently of the PI3-K-Akt and the JNK-c-Jun pathways in sympathetic neurons

**Figure 1:**
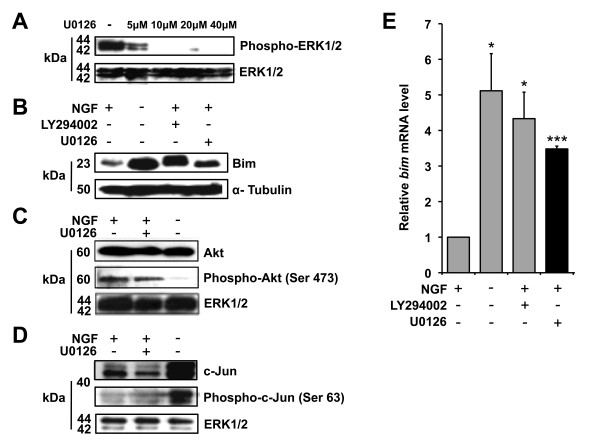
**The MEK-ERK pathway negatively regulates *bim *mRNA expression in sympathetic neurons**. (A) Effect of U0126 on phosphorylation of ERK1/2 in sympathetic neurons. Immunoblots were performed with whole cell extracts from sympathetic neurons maintained in the presence of NGF and left untreated or treated with U0126 at 5, 10, 20 or 40 μM for 16 hours. Antibodies to detect total ERK1/2 (Cell Signaling #9102) and phospho-ERK1/2 (Cell Signaling #9101) were used. (B) Effect of U0126 or LY294002 on Bim protein levels in sympathetic neurons. Immunoblots were performed with whole cell extracts from sympathetic neurons maintained in the presence of NGF, withdrawn from NGF for 16 hours, or treated with 50 μM LY294002, or with 10 μM U0126, for 16 hours in the presence of NGF. Antibodies to detect Bim (Chemicon AB17003) and α-Tubulin (AbD Serotec MCA77G) were used. (C) U0126 does not alter Akt phosphorylation in sympathetic neurons. Immunoblots were performed with whole cell extracts from sympathetic neurons maintained in the presence of NGF, treated with 10 μM U0126 for 16 hours in the presence of NGF, or withdrawn from NGF for 16 hours. Antibodies to detect total Akt (Cell Signaling #9272), phospho-Akt (Cell Signaling #9271) and total ERK1/2 (Cell Signaling #9102) were used. (D) U0126 does not alter c-Jun phosphorylation in sympathetic neurons. Cells were treated as in (C) and antibodies to detect total c-Jun (BD Biosciences #610327), phospho-c-Jun (Santa Cruz sc-822) and total ERK1/2 (Cell Signaling #9102) were used. (E) Total RNA was prepared from sympathetic neurons treated as in (B) and *bim *mRNA levels were analysed by q-PCR relative to the level of the mRNA encoded by the house-keeping gene *Hprt1*. The data is presented as the mean ± S.E., n = 3. Endogenous *bim *mRNA levels increased significantly following NGF withdrawal and treatment with LY294002, respectively (p = 0.015 and p = 0.014). Treatment with U0126 also induced a significant increase in the level of *bim *mRNA (p = 0.0003).

To investigate whether the MEK-ERK signalling pathway regulates the steady state level of *bim *mRNA in sympathetic neurons cultured in the presence of NGF, we used a well characterised MEK inhibitor, U0126, to reduce MEK and therefore ERK activity. To confirm that phospho-ERK1/2 levels are reduced when sympathetic neurons are treated with U0126, immunoblots were performed with extracts from sympathetic neurons either maintained in NGF-containing medium or treated with increasing concentrations of U0126 in the presence of NGF (Figure 1A). Treatment of sympathetic neurons with U0126 at 10 μM strongly reduced the phosphorylation of ERK1 and ERK2, whereas total ERK protein levels were not altered (Figure 1A).

Next, we verified that Bim protein levels are upregulated in sympathetic neurons following treatment with U0126 or with the PI3-K inhibitor LY294002 (Figure 1B). Immunoblots were performed using extracts from sympathetic neurons either maintained in NGF-containing medium, withdrawn from NGF for 16 hours or treated with either LY294002 (50 μM) or U0126 (10 μM) in the presence of NGF for 16 hours. Bim protein levels increased strongly following NGF withdrawal and following treatment with U0126 or with LY294002, whereas α-Tubulin protein levels were not altered (Figure 1B).

We then confirmed that U0126 inhibits MEK-ERK signalling without affecting the two major signalling pathways known to be involved in Bim regulation, the PI3-K-Akt pathway and the JNK-c-Jun pathway (Figure 1C, D). Immunoblots were performed with extracts from sympathetic neurons either maintained in the presence of NGF, withdrawn from NGF for 16 hours or treated with U0126 (10 μM) for 16 hours in the presence of NGF. As expected, we found that the level of phosphorylation of Akt at Ser 473 is high when neurons are maintained in the presence of NGF and falls following NGF withdrawal [8] (Figure 1C) and that c-Jun phosphorylation at serine 63 increases following NGF withdrawal [19] (Figure 1D). Importantly, treatment with U0126 did not affect phospho-Akt or phospho-c-Jun levels (Figure 1C, D), thereby confirming that the PI3-K-Akt and the JNK-c-Jun pathways are independent of MEK-ERK signalling in sympathetic neurons.

To investigate whether the MEK-ERK pathway regulates *bim *mRNA levels, we carried out q-PCR with cDNA prepared from sympathetic neurons maintained in NGF-containing medium, withdrawn from NGF for 16 hours, or treated with either LY294002 (50 μM) or U0126 (10 μM) in the presence of NGF for 16 hours (Figure 1E). The level of *bim *mRNA was analysed relative to the level of the transcripts for the house-keeping genes *Hprt1 *and *Gapdh. Bim *mRNA levels relative to *Hprt1 *are shown (Figure 1E), since both house-keeping genes behaved in a similar way. After NGF withdrawal, the level of *bim *mRNA increased by 5 fold and upon treatment with LY294002 it increased by 4.2 fold, as described previously [8]. Interestingly, when the cells were treated with U0126, there was also a significant (3.4 fold) increase in the level of *bim *mRNA. This data indicates that in the presence of NGF the MEK-ERK pathway negatively regulates *bim *mRNA expression in sympathetic neurons.

### The MEK-ERK pathway negatively regulates *bim *mRNA expression in sympathetic neurons via regulatory elements outside of the *bim *promoter, exon 1 and first intron

**Figure 2:**
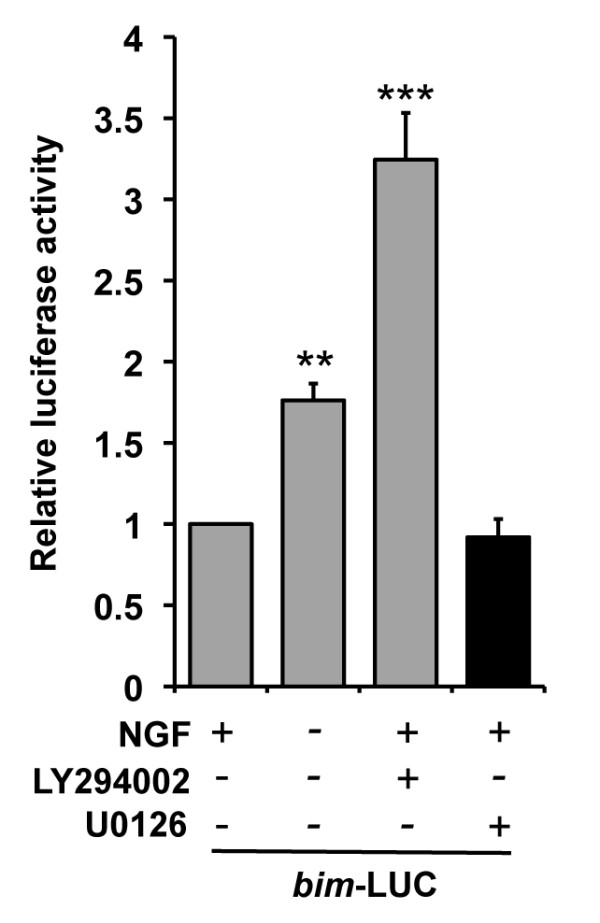
**The MEK-ERK pathway negatively regulates *bim *expression via regulatory elements outside of the *bim *promoter, exon 1 and intron 1**. Sympathetic neurons were microinjected with the *bim*-LUC construct (10 ng/μl) along with pRL-TK (5 ng/μl) to control for injection efficiency. The cells were either maintained in medium containing NGF, withdrawn from NGF, treated with LY294002 at 50 μM, or with U0126 at 10 μM, in the presence of NGF. After 16 hours, luciferase activity was measured. Relative luciferase activity was determined by normalisation of *Firefly *luciferase activity to *Renilla *luciferase activity (pRL-TK refers to *Renilla *luciferase under the control of the thymidine kinase promoter). The data is presented as the mean ± S.E., n = 4. *Bim*-LUC is activated significantly following NGF withdrawal or after treatment with LY294002, respectively (p = 0.003 and p = 0.001). However, treatment with U0126 does not activate the *bim*-LUC construct.

To determine the mechanism by which the MEK-ERK pathway negatively regulates *bim *expression in sympathetic neurons we investigated which region of the *bim *gene mediates this effect. Initially, sympathetic neurons were microinjected with a *bim*-LUC reporter construct to determine whether there are any MEK-ERK-responsive elements within the 5.2 kb fragment of *bim *that is cloned in *bim*-LUC. This construct contains 2.5 kb of the *bim *promoter, the non-coding exon 1 and the 2.5 kb first intron [8]. Following injection, the cells were either maintained in medium containing NGF, withdrawn from NGF, or treated with either LY294002 (50 μM) or U0126 (10 μM) in the presence of NGF, and luciferase activity was determined after 16 hours (Figure 2). Following NGF withdrawal, or treatment with LY294002 [8], *bim-*LUC was activated significantly (Figure 2). However, when the cells were treated with U0126 there was no increase in the activity of *bim*-LUC (Figure 2). This suggests that there are no MEK-ERK-responsive elements within the first 2.5 kb of the *bim *promoter, exon 1 or the first intron.

### The *bim *3' UTR contains elements that are responsive to NGF withdrawal

**Figure 3:**
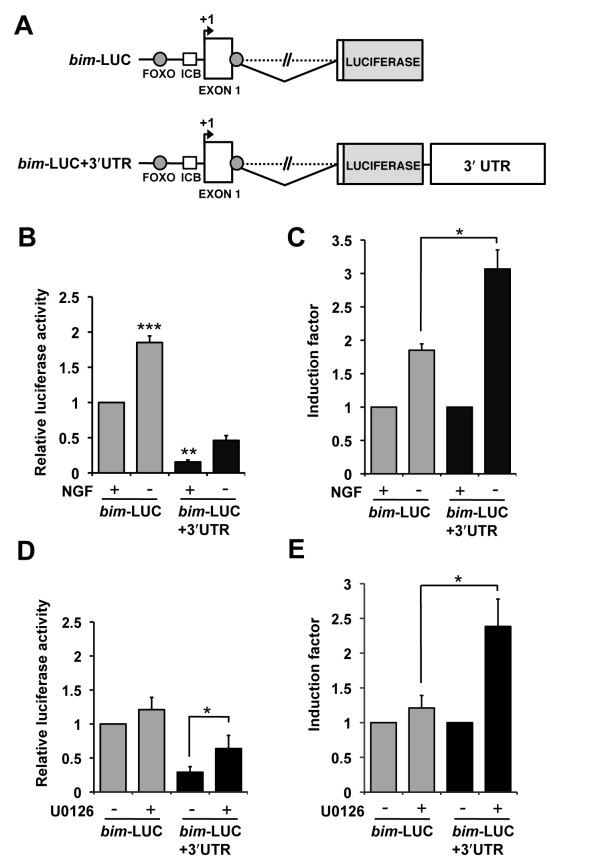
**The MEK-ERK pathway negatively regulates *bim *expression through the *bim *3'UTR**. (A) Structure of the *bim*-LUC and *bim*-LUC+3'UTR reporter constructs. *Bim*-LUC+3'UTR was constructed by cloning and inserting the 4.2 kb rat *bim *3'UTR into the *bim*-LUC construct [8], upstream of the SV40 late poly (A) signal and downstream of the luciferase reporter gene. The locations of two conserved FOXO binding sites and an inverted CCAAT box (ICB) are shown. (B) Sympathetic neurons were microinjected with *bim*-LUC at 10 ng/μl or *bim*-LUC+3'UTR at 14 ng/μl, along with pRL-TK to control for injection efficiency (5 ng/μl). The cells were maintained in medium containing NGF (+NGF) or withdrawn from NGF (-NGF) for 16 hours, after which time luciferase activity was measured. Relative luciferase activity was determined by normalisation of *Firefly *luciferase activity to *Renilla *luciferase activity (pRL-TK refers to *Renilla *luciferase under the control of the thymidine kinase promoter). The data is presented as the mean ± S.E., n = 5. *Bim*-LUC is activated significantly following NGF withdrawal (p = 0.001). Addition of the *bim *3' UTR significantly decreases the basal promoter level of *bim*-LUC (p = 0.003). (C) The basal levels of *bim*-LUC and *bim*-LUC+3'UTR were normalised to 1 and the induction factors of the two constructs were compared. Addition of the *bim *3' UTR significantly increases the induction of *bim*-LUC following NGF withdrawal (p = 0.018). (D) Sympathetic neurons were microinjected with *bim*-LUC at 10 ng/μl or *bim*-LUC+3'UTR at 14 ng/μl, along with pRL-TK (5 ng/μl). The cells were maintained in medium containing NGF and either left untreated (-U0126) or treated with U0126 at 10 μM (+U0126) for 16 hours, after which time luciferase activity was measured. The data is presented as the mean ± S.E., n = 6. *Bim*-LUC+3'UTR is activated significantly following inhibition of the MEK-ERK pathway by treatment with U0126 (p = 0.015). *Bim*-LUC is not activated significantly following treatment with U0126. (E) The basal levels of *bim*-LUC and *bim*-LUC+3'UTR were normalised to 1 and the induction factors of the two constructs were compared. Addition of the *bim *3' UTR significantly increases the induction of *bim*-LUC upon inhibition of the MEK-ERK pathway (p = 0.022).

Our results indicate that the region that mediates the regulation of *bim *by the MEK-ERK pathway is not located at the 5' end of the *bim *gene. Therefore we hypothesised that the *bim *3' UTR may contain the target region, since the 3' UTR of a gene often contains a number of regulatory motifs that are important for modulating gene expression. These can include transcriptional enhancers or silencers, or sequences in the 3' UTR of the mRNA that are targeted by microRNAs or bound by RNA binding proteins that regulate mRNA stability. Initially, we mapped the 3' end of the rat *bim *mRNA by 3' RACE. This indicated that the 3' UTR is 4.2 kb long. We then cloned the entire *bim *3' UTR into our *bim*-LUC reporter construct, downstream of the luciferase gene, to generate the *bim*-LUC+3'UTR construct (Figure 3A). To compare the *bim*-LUC+3'UTR construct to *bim*-LUC, sympathetic neurons were microinjected with equimolar concentrations of *bim*-LUC+3'UTR or *bim*-LUC, and either maintained in the presence of NGF or withdrawn from NGF for 16 hours, after which time luciferase activity was determined (Figure 3B, C). We found that the addition of the 3' UTR greatly reduced the basal level of the *bim*-LUC construct in the presence of NGF, suggesting that the *bim *3' UTR contains sequences that reduce luciferase activity at the level of transcription, RNA stability or translation. Critically, addition of the 3' UTR to the *bim*-LUC reporter significantly increased its induction from 1.8 fold to 3 fold, following NGF withdrawal (Figure 3C). This demonstrates that the *bim *3' UTR contains elements that are responsive to NGF withdrawal. It is likely that the new *bim*-LUC+3'UTR construct is more representative of the endogenous *bim *gene than the original *bim*-LUC construct since endogenous *bim *mRNA levels increase by around 5 fold after NGF withdrawal (Figure 1E).

### The *bim *3'UTR is a target of the MEK-ERK pathway in sympathetic neurons

To ascertain whether the *bim *3' UTR is a target of the MEK-ERK pathway, sympathetic neurons were microinjected with *bim*-LUC+3'UTR or *bim*-LUC and the cells were either maintained in medium containing NGF or treated with U0126 (10 μM) in the presence of NGF. After 16 hours, relative luciferase activity was determined (Figure 3D, E). As demonstrated previously, inhibition of the MEK-ERK pathway with U0126 did not activate the *bim*-LUC construct (Figure 3D, E). However, when *bim*-LUC+3'UTR was treated with U0126 there was a significant activation of the reporter to 2.5 fold (Figure 3D, E). This suggests that the regulation of *bim *mRNA level via the MEK-ERK-pathway is mediated by the 3' UTR region of the *bim *gene.

### Inhibition of the MEK-ERK pathway does not alter *bim *mRNA stability

**Figure 4:**
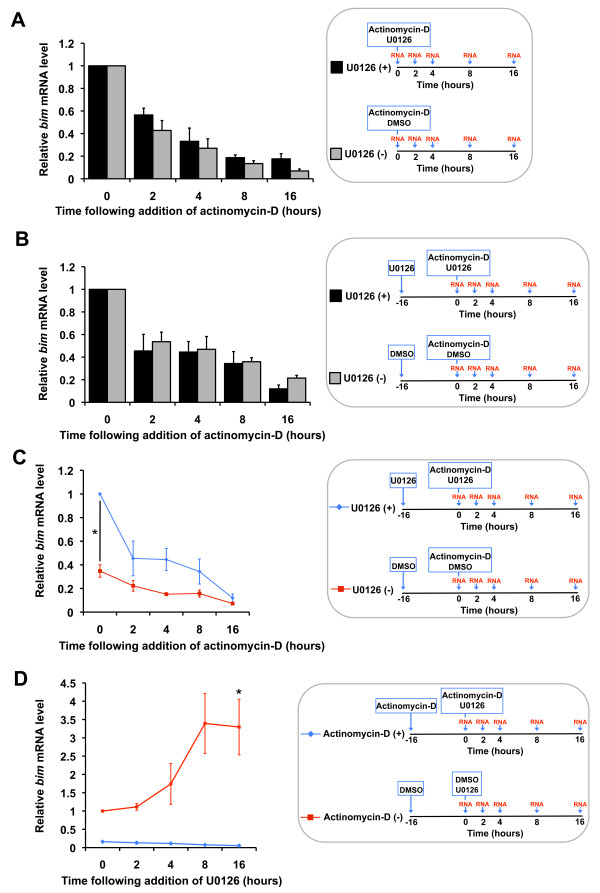
**U0126 does not alter *bim *mRNA stability in sympathetic neurons**. (A) Sympathetic neurons were treated with actinomycin-D at 0.1 μg/ml in the presence of NGF. The cells were either maintained in medium containing NGF (-U0126) or treated with U0126 at 10 μM in the presence of NGF (+U0126). Actinomycin-D and U0126 were added to the cells together and then total RNA was isolated at time point 0 hours, 2 hours, 4 hours, 8 hours and 16 hours. The level of *bim *mRNA was analysed by q-PCR relative to the level of the mRNAs encoded by the house-keeping genes *Hprt1 *and *Gapdh*. *Bim *mRNA levels relative to *Hprt1 *are shown here. The data is presented as the mean ± S.E., n = 5. There is no significant difference in the stability of *bim *mRNA in sympathetic neurons treated with or without U0126 over 16 hours. (B) Sympathetic neurons were treated as in (A) but with the following modification: U0126 was added to the cells for 16 hours prior to the addition of actinomycin-D and U0126 together (for +U0126 conditions). Following addition of actinomycin-D, total RNA was isolated at time point 0 hours, 2 hours, 4 hours, 8 hours and 16 hours. The level of *bim *mRNA was analysed by q-PCR relative to the house-keeping genes *Hprt1 *and *Gapdh*. *Bim *mRNA levels relative to *Hprt1 *are shown here. The data is presented as the mean ± S.E., n = 3. There is no significant difference in the stability of *bim *mRNA in sympathetic neurons treated with or without U0126 over 16 hours. (C) Q-PCR data from (B) with raw *bim *mRNA levels at timepoint 0 (+U0126 and -U0126 *bim *mRNA levels have not been normalised to 1). Treatment with U0126 for 16 hours elevates *bim *mRNA levels significantly (p = 0.021), but does not significantly alter the half life of the *bim *mRNA. (D) Sympathetic neurons were either maintained in medium containing NGF (-actinomycin-D) or treated with actinomycin-D (+actinomycin-D) at 0.1 μg/ml in the presence of NGF. After 16 hours, the cells were treated with U0126 (together with actinomycin-D for +actinomycin-D conditions) and then total RNA was isolated at time point 0 hours, 2 hours, 4 hours, 8 hours and 16 hours. The level of *bim *mRNA was analysed by q-PCR relative to *Hprt1 *and *Gapdh. Bim *mRNA levels relative to *Hprt1 *are shown here. The data is presented as the mean ± S.E., n = 3. Treatment with U0126 for 16 hours, in the absence of actinomycin-D, elevates *bim *mRNA levels significantly (p = 0.036).

Since the MEK-ERK-responsive elements are located within the *bim *3' UTR, we decided to investigate whether U0126 alters *bim *mRNA stability. We used actinomycin-D to inhibit *bim *transcription and measured *bim *mRNA levels over a time course with or without U0126 (Figure 4). In the first set of experiments actinomycin-D (0.1 μg/ml) and U0126 (10 μM) were added to the cells together at time point 0, and then total RNA was isolated at time point 0 hours to 16 hours, as 16 hours is the time point at which the original effect of U0126 was observed (Figure 1E; 4A). The level of *bim *mRNA was analysed by q-PCR relative to the level of the transcripts for the house-keeping genes *Hprt1 *and *Gapdh*. *Bim *mRNA levels relative to *Hprt1 *are shown (Figure 4), since both house-keeping genes behaved in a similar way - neither was affected by the addition of U0126 or actinomycin-D. Following the addition of actinomycin-D, *bim *mRNA decayed over the 16-hour time course in the absence of U0126 (Figure 4). The half-life of rat *bim *mRNA is between 2 and 4 hours in NGF-maintained sympathetic neurons treated with actinomycin-D (Figure 4A, B). Importantly, the addition of U0126 did not significantly increase the stability of the *bim *mRNA over the 16 hour time course (Figure 4A). To be sure that this was a representative result, we performed a second experiment in which we pre-treated sympathetic neurons with U0126 for 16 hours prior to the addition of actinomycin-D (for +U0126 conditions) (Figure 4B, C). This would therefore rule out the possibility that actinomycin-D was interfering with the activity of U0126. However, the same result was obtained with no significant difference in the half life of the *bim *mRNA in sympathetic neurons treated with or without U0126 (Figure 4B). This indicates that U0126 does not increase *bim *mRNA levels by altering mRNA stability, in sympathetic neurons.

As a further control, we included a time course q-PCR experiment in which we compared cells treated with U0126 and actinomycin-D to those treated with U0126 only (Figure 4D). We found that *bim *mRNA levels increased over the 16 hour time course and were significantly higher, 3.3 fold, at 16 hours following treatment with U0126 compared to time point 0, and that treatment with actinomycin-D abolished this up-regulation (Figure 4D). Furthermore, this data corroborates our initial finding that treatment with U0126 for 16 hours significantly elevates the level of *bim *mRNA in sympathetic neurons (Figure 1E,4D).

### The MEK1/2-ERK1/2 pathway negatively regulates *bim *mRNA expression in sympathetic neurons

**Figure 5:**
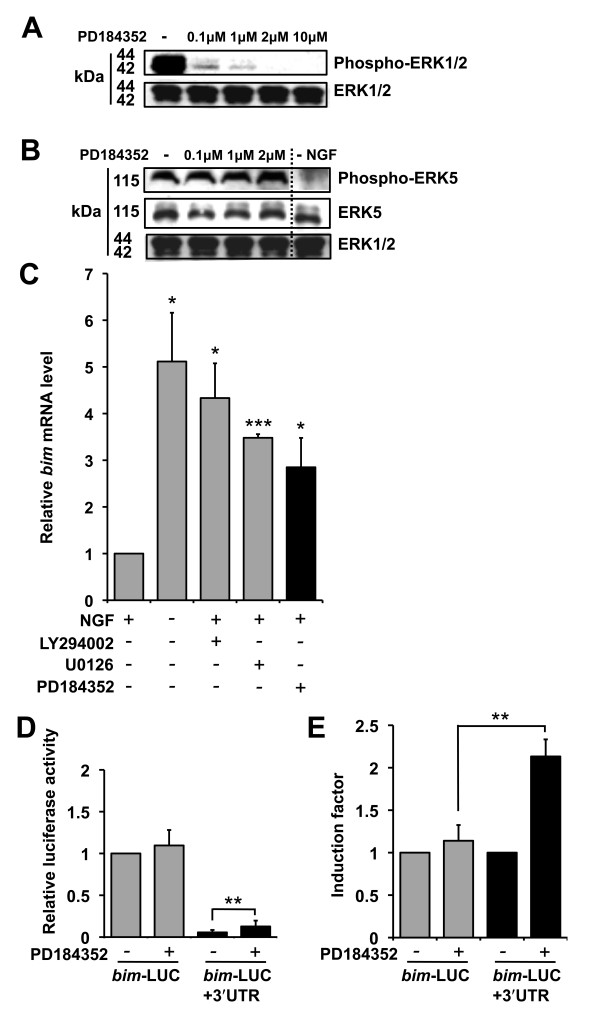
**The MEK1/2-ERK1/2 pathway negatively regulates *bim *mRNA expression in sympathetic neurons**. (A) Effect of PD184352 on phosphorylation of ERK1/2 in sympathetic neurons. Immunoblots were performed with whole cell extracts from sympathetic neurons maintained in the presence of NGF and left untreated or treated with PD184352 at 0.1, 1, 2 or 10 μM for 16 hours. Antibodies to detect total ERK1/2 (Cell Signaling #9102) and phospho-ERK1/2 (Cell Signaling #9101) were used. (B) Effect of PD184352 on phosphorylation of ERK5 in sympathetic neurons. Cells were treated as in (A), but including a set of extracts prepared from cells withdrawn from NGF for 16 hours, and antibodies to detect total ERK5 (Cell Signaling #3372S) and phospho-ERK5 (Santa Cruz sc16564) were used. (C) Q-PCR data re-plotted from Figure 1E, but including the final data group where neurons were treated with PD184352 at 2 μM for 16 hours in the presence of NGF. The data is presented as the mean ± S.E., n = 3. Endogenous *bim *mRNA levels increased significantly following NGF withdrawal (p = 0.015), treatment with LY294002 or U0126, respectively (p = 0.014 and p = 0.0003). Treatment with PD184352 also induced a significant increase in the level of *bim *mRNA (p = 0.05). (D) Sympathetic neurons were microinjected with *bim*-LUC at 10 ng/μl or *bim*-LUC+3'UTR at 14 ng/μl, along with pRL-TK (5 ng/μl). The cells were maintained in medium containing NGF and either left untreated (-PD184352) or treated with PD184352 at 2 μM (+PD184352) for 16 hours, after which time luciferase activity was measured. Relative luciferase activity was determined by normalisation of *Firefly *luciferase activity to *Renilla *luciferase activity (pRL-TK refers to *Renilla *luciferase under the control of the thymidine kinase promoter). The data is presented as the mean ± S.E., n = 4. *Bim*-LUC+3'UTR is activated significantly following inhibition of the MEK1/2-ERK1/2 pathway by treatment with PD184352 (p = 0.005). *Bim*-LUC is not activated significantly following treatment with PD184352. (E) The basal levels of *bim*-LUC and *bim*-LUC+3'UTR were normalised to 1 and the induction factors of the two constructs were compared. Addition of the *bim *3' UTR significantly increases the induction of *bim*-LUC upon inhibition of the MEK1/2-ERK1/2 pathway (p = 0.009).

To investigate which of the MEK-ERK signalling pathways, MEK1/2-ERK1/2 or MEK5-ERK5, regulates *bim *mRNA levels in sympathetic neurons we used a specific MEK1/2 inhibitor, PD184352 [20]. To confirm that phospho-ERK1/2 levels are reduced when sympathetic neurons are treated with PD184352, immunoblots were performed with extracts from sympathetic neurons either maintained in NGF-containing medium or treated with increasing concentrations of PD184352 in the presence of NGF (Figure 5A). Treatment of sympathetic neurons with PD184352 at 2 μM strongly reduced the phosphorylation of ERK1 and ERK2, whereas total ERK protein levels were not altered (Figure 5A). To ensure that PD184352 specifically inhibits MEK1/2, and does not interfere with MEK5-ERK5 signalling in sympathetic neurons, we repeated immunoblots on the PD184352-treated samples (including a set of extracts prepared from cells withdrawn from NGF for 16 hours) with antibodies that detect phospho-ERK5 and ERK5 (Figure 5B). NGF withdrawal reduced phosphorylation of ERK5 and, as expected treatment of sympathetic neurons with PD184352 at 2 μM in the presence of NGF did not affect the phosphorylation of ERK5 and total ERK 5 levels were not altered either (Figure 5B).

We then performed q-PCR, as for Figure 1E, including cDNA prepared from sympathetic neurons treated with PD184352 at 2 μM in the presence of NGF for 16 hours (Figure 5C). The level of *bim *mRNA was analysed relative to the level of the transcripts for the house-keeping genes *Hprt1 *and *Gapdh *(*Bim *mRNA levels relative to *Hprt1 *are shown). When sympathetic neurons were treated with PD184352 there was a significant (2.9 fold) increase in the level of *bim *mRNA (Figure 5C). This was a similar fold induction to that observed following treatment with U0126 (Figure 1E, 5C).

Next, we microinjected sympathetic neurons with *bim*-LUC+3'UTR or *bim*-LUC and the cells were either maintained in medium containing NGF or treated with PD184352 (2 μM) in the presence of NGF. After 16 hours, relative luciferase activity was determined (Figure 5D, E). Inhibition of the MEK1/2-ERK1/2 pathway with PD184352 did not activate the *bim*-LUC construct, but significantly activated the *bim*-LUC+3'UTR construct by 2.1 fold (Figure 5D, E). Taken together, this data confirms that the MEK1/2-ERK1/2 pathway represses *bim *mRNA levels through the *bim *3'UTR.

### The MEK-ERK pathway contributes to cell survival in sympathetic neurons

**Figure 6:**
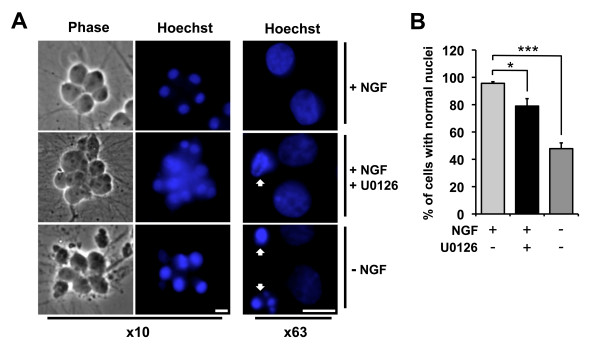
**Effect of U0126 on cell viability in sympathetic neurons**. Sympathetic neurons were either maintained in the presence of NGF, withdrawn from NGF or treated with U0126 (10 μM) in the presence of NGF. The cells were fixed and stained with Hoechst dye and normal and pyknotic nuclei were counted at time point 0, 24, 48 and 72 hours. (A) Example images of sympathetic neurons maintained in the presence of NGF, treated with U0126 for 72 hours or withdrawn from NGF for 72 hours. Neurons were considered viable if they retained a normal nuclear morphology. The white arrows indicate dead cells. The bar represents 10 μm. (B) Effect of U0126 on cell viability at 72 hours. The data is presented as the mean ± S.E., n = 3. Treatment with U0126 (p = 0.040) and NGF withdrawal (p = 0.0004) significantly decreases the percentage of viable neurons at 72 hours compared to the cells that were maintained in the presence of NGF.

Finally, to determine whether inhibition of the prosurvival MEK-ERK pathway is sufficient to induce cell death in sympathetic neurons we studied the effect of U0126 on cell viability over 72 hours (Figure 6). Sympathetic neurons were either maintained in the presence of NGF, withdrawn from NGF or treated with U0126 (10 μM) in the presence of NGF. The cells were then fixed and stained with Hoechst dye to visualise the nuclei and the number of cells with normal or pyknotic nuclei was determined at time point 0, 24, 48 and 72 hours. Viable cells retained a normal nuclear morphology and were not pyknotic (Figure 6A, +NGF). At 72 hours, the percentage of viable cells maintained in the presence of NGF was over 95% (Figure 6B), whereas only 78% of the cells treated with U0126 had normal nuclei (Figure 6B). This data indicates that the MEK-ERK pathway independently contributes to cell survival in NGF-treated sympathetic neurons.

## Discussion

**Figure 7:**
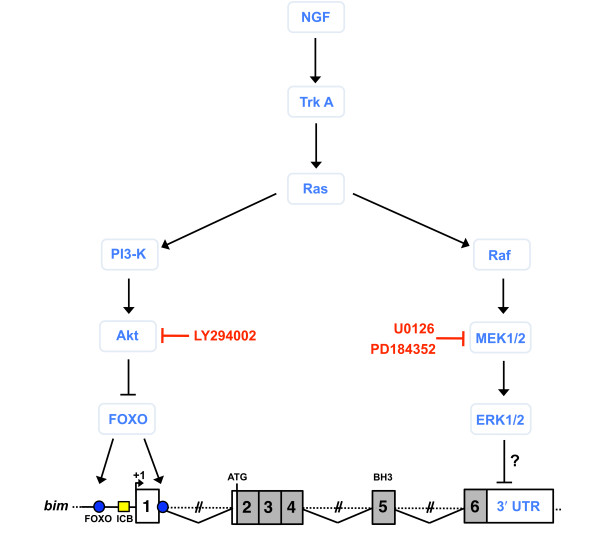
**Model of the transcriptional regulation of *bim *expression by prosurvival signalling pathways in sympathetic neurons**. The MEK-ERK pathway negatively regulates *bim *expression at the level of transcription via the 3' UTR by an unknown mechanism. PI3-K-Akt signalling inhibits FOXO transcription factors which can bind to two conserved FOXO sites in the *bim *promoter [8,9]. Both prosurvival pathways are activated following the binding of NGF to TrkA and act independently of each other to reduce *bim *mRNA levels.

By using the MEK inhibitor U0126, we have demonstrated that the prosurvival MEK-ERK pathway represses *bim *mRNA levels in sympathetic neurons through the 3' UTR. This effect is independent of PI3-K-Akt signalling and represses *bim *transcription in the presence of NGF (Figure 7).

After initially observing that the MEK-ERK pathway negatively regulates *bim *mRNA expression in sympathetic neurons, we generated a *bim*-LUC+3'UTR reporter construct to investigate a potential role for the 3' UTR in this mechanism. By microinjecting the *bim*-LUC+3'UTR construct into sympathetic neurons, we localised the region responsive to MEK-ERK signalling to the *bim *3' UTR. In addition, we found that the *bim*-LUC+3'UTR construct is a useful tool for studying *bim *regulation since it has a lower basal expression in the presence of NGF and an increased induction following NGF withdrawal. It may therefore be more representative of the endogenous *bim *gene, in comparison with our previous *bim*-LUC reporter [8,9].

To further investigate this mechanism, we studied *bim *mRNA stability following treatment with or without U0126. Since we found no significant effect upon the half life of the *bim *mRNA following treatment with U0126, we concluded that MEK/ERK signalling does not affect *bim *mRNA stability, and therefore may regulate *bim *mRNA levels by a transcriptional mechanism. Future studies will uncover potential cis-acting elements located in the *bim *3' UTR, such as enhancers or silencers that may bind DNA-binding proteins that regulate transcription from the upstream *bim *promoter [21]. In principle, these regulators might be activators that are repressed by MEK-ERK signalling, or alternatively they could be repressors that are activated by the MEK-ERK pathway.

Recently, other groups have reported a role for the *bim *3' UTR in modulating *bim *mRNA levels. Studies with Baf-3 cells have revealed that *bim *mRNA is stabilised when IL3 is limiting and this regulation occurs via AU-rich elements found within the *bim *3' UTR [22]. *Bim *is also a target for regulation by miRNAs: the *bim *3' UTR contains sequences targeted by miR-17~92 microRNAs and the conditional knockout of miR-17~92 results in elevated Bim levels and an inhibition of B cell development [23]. The *bim *mRNA can also be targeted by miR-221 and miR-222, both of which are induced by NGF-stimulation in PC12 cells and this is linked to the MEK1/2-ERK1/2 pathway [24]. However, all of these studies are associated with mRNA stability and not transcription, so is unlikely that any of them account for the mechanism we have described here.

In sympathetic neurons both the MEK1/2-ERK1/2 and the MEK5-ERK5 signalling pathways are stimulated in response to NGF and are activated downstream of Ras [25], and U0126 inhibits both MEK1/2 and MEK5 [26]. A number of reports have described the importance of MEK1/2-ERK1/2 signalling in the post-translational control of the Bim protein in different cell types [13-18]. The downstream events involved in MEK5-ERK5 signalling in neurons are less well defined, but it has been shown that ERK5 can play an important role in neurotrophin-mediated survival, and deletion of the *erk5 *gene results in a significant increase in apoptosis in developing sympathetic neurons [27]. To determine which of the ERK signalling pathways targets the 3' UTR of the *bim *gene, we used the specific MEK1/2-ERK1/2 inhibitor PD184352 [20]. We found that this compound had similar effects to U0126, indicating that this mechanism of *bim *regulation largely depends on the MEK1/2-ERK1/2 pathway in sympathetic neurons.

Finally, we showed that inhibition of the prosurvival MEK-ERK pathway by treatment with U0126 was sufficient to induce some neuronal death in the presence of NGF. However, it should be noted that the effect was modest compared to that observed following NGF deprivation. This is probably because the PI3-K-Akt survival pathway is still active in cells treated with U0126 (Figure 1).

## Conclusions

It is now evident that a number of complex regulatory mechanisms are in place to constrain the expression and the activity of the proapoptotic Bim protein. Thus, using the well-characterised model of NGF-dependent developing sympathetic neurons, we have identified an additional mechanism by which *bim *mRNA levels are regulated. We have shown that MEK-ERK signalling represses *bim *mRNA levels through the *bim *3' UTR (Figure 7). Since other reports investigating the function of the *bim *3' UTR in modulating *bim *mRNA expression have focused on the role of mRNA stability, it is likely that the mechanism we have identified here has not yet been studied in other cell types.

## Methods

### Plasmid constructs

The *bim*-LUC reporter construct consists of a 5.2 kb fragment, containing the region immediately 5' to the rat *bim *initiator codon, sub-cloned into pGL3-Basic [8]. The *bim*-LUC+3'UTR reporter construct was generated by PCR amplification of the rat *bim *3' UTR from a restriction fragment cloned from the PAC clone 215h9 (rat P1 artificial chromosome (PAC) library RPC131, UK Human Genome Mapping Project Resource Centre, Cambridge, UK). Initially, 3' RACE was used to define the end point of the rat *bim *3' UTR for subsequent cloning: RACE was performed on rat lung poly A+ RNA using the Marathon cDNA Amplification Kit (Clontech). The following primers were then used to generate the 3'UTR in 2 fragments: Fragment 1, 5'-CTCACTAGTCAGGAGCTTCGTGCAG-3' and 5'-CTCCTACAAGGCACAAAACCCG-3'; fragment 2, 5'-CTATACGGATGTCCCTGTACTGTATC-3' and 5'-CTCACTAGTCATGAGAGCTAGTCGCAA-3'. The 3' UTR was assembled in pBluescript SK (Stratagene) using a unique BglII restriction site within the 4.2-kb region to link fragments 1 and 2. The 3' UTR was sub-cloned into *bim*-LUC, using an XbaI restriction site downstream of the luciferase reporter gene and upstream of the SV40 late poly (A) signal.

### Cell culture

Sympathetic neurons were isolated from the superior cervical ganglia (SCG) of 1-day-old Sprague Dawley rats and cultured as described previously [28]. Animal experiments were performed according to the Animals (Scientific Procedures) Act 1986 under a license reviewed and approved by the Biological Services Unit at University College London. Cells were maintained in SCG medium supplemented with 2.5S NGF (Cedarlane) at 50 ng/ml, and fluorodeoxyuridine and uridine (both from Sigma-Aldrich) each at 20 μM. In NGF withdrawal experiments, cells were rinsed twice with medium (without NGF) and then re-fed with medium containing an anti-NGF antibody at 100 ng/ml (Chemicon). The PI3-K inhibitor LY294002 was used at a concentration of 50 μM and the MEK inhibitor U0126 at a concentration of 10 μM (both from Promega). The MEK1/2 inhibitor PD184352 was used at a concentration of 2 μM and was kindly provided by Simon Cook (The Babraham Institute). The RNA synthesis inhibitor actinomycin-D was used at a concentration of 0.1 μg/ml. When actinomycin-D was used in conjunction with U0126, cells were either pre-treated with U0126 (or actinomycin-D) for 16 hours prior to the addition of actinomycin-D (or U0126) and then actinomycin-D and U0126 were added at the same time (time point 0). LY294002, U0126 and PD184352 were dissolved in DMSO and therefore equal volumes of DMSO were added to the untreated cells as a control.

### Microinjection

Sympathetic neurons were cultured *in vitro *for 5-7 days and then microinjected as described previously [3]. Dual luciferase assays were carried out on injected cells using the Dual Luciferase reporter assay system (Promega). Cells were harvested for luciferase assays at 16 hours following NGF withdrawal.

### Real-time quantitative PCR (q-PCR)

RNA was isolated from sympathetic neurons and reverse transcribed as described above. A *bim *specific primer and probe set was designed using the PrimerExpress software v2.0 (Applied Biosystems): *bim *forward primer 5'-CCAGGCCTTCAACCATTATCTC-3', *bim *reverse primer 5'-GCGCAGATCTTCAGGTTCCT-3' and *bim *probe 5'-TGCAATGGCTTCCATAAGGCCAGTCTCA-3'. *Glyceraldehyde-3-phosphate dehydrogenase *(*Gapdh*) and *Hypoxanthine phosphoribosyltransferase 1 *(*Hprt1*) were used as house-keeping (control) genes (both from Applied Biosystems). Q-PCR data was analysed using the 2^-ΔΔCT ^relative quantitation method [29].

### Immunoblotting

Immunoblotting was carried out with whole cell extracts from sympathetic neurons as described previously [30]. Proteins were separated on 7% or 12% SDS polyacrylamide gels. The following rabbit polyclonal primary antibodies from Cell Signaling were used: ERK1/2 antibody (#9102), phospho-ERK1/2 (Thr 202/Tyr 204) (#9101), ERK5 (#3372S), Akt antibody (#9272) and phospho-Akt antibody (Ser 473) (#9271). The phospho-ERK5 (Thr 218/Tyr 220) goat polyclonal antibody was from Santa Cruz (sc-16564), as was the mouse monoclonal phospho-c-Jun (Ser 63) antibody (sc-822). The mouse monoclonal c-Jun antibody was from BD Biosciences (#610327), the rabbit polyclonal Bim antibody was from Chemicon (AB17003) and the rat monoclonal α-Tubulin antibody was from AbD Serotec. For each immunoblotting experiment, several repeats were carried out and representative blots are shown.

### Immunocytochemistry

Sympathetic neurons were fixed in 4% paraformaldehyde, permeabilised in 0.5% Triton-X-100, and stained with Hoechst dye to visualise nuclear morphology. Slides were viewed using a Zeiss Axioplan 2 fluorescence microscope using a Plan-Apochromat ×10 objective or ×63 oil objective. Cells were scored in a blinded manner wherever possible.

### Statistical analysis

In each set of experiments data is normalised to a control sample (for example, *bim*-LUC +NGF is set to 1). For microinjection, the relative induction of a DNA construct is calculated by dividing the relative luciferase activity in the absence of NGF by the relative luciferase activity in the presence of NGF. All data are presented as the mean ± S.E. of multiple experiments.

The statistical significance of differences between means was evaluated by performing an unpaired Student's T-test (for two-tailed distributions). To compare normalised data to the control sample, that has no error associated to it (for example, when comparing data to *bim*-LUC +NGF, which is set to 1), the log10 values of the data were taken and a one sample T-test was used (for two-tailed distributions). Statistical significance is presented as p-values: *** p < 0.001, ** p < 0.01, * and p < 0.05.

## Authors' contributions

JG and MK carried out immunoblots and edited the paper. RH performed all of the remaining experiments and wrote the paper. JH designed the study and wrote the paper. All authors read and approved the final manuscript.
